# Carbon Dioxide Emissions and Methane Flux from Forested Wetland Soils of the Great Dismal Swamp, USA

**DOI:** 10.1007/s00267-019-01177-4

**Published:** 2019-06-25

**Authors:** Laurel Gutenberg, Ken W. Krauss, John J. Qu, Changwoo Ahn, Dianna Hogan, Zhiliang Zhu, Chenyang Xu

**Affiliations:** 1 George Mason University, 4400 University Dr., Fairfax, VA 22030 USA; 2 U.S. Geological Survey, Wetland and Aquatic Research Center, 700 Cajundome Blvd., Lafayette, LA 70506 USA; 3 U.S. Geological Survey, 12201 Sunrise Valley Dr., Reston, VA 20192 USA

**Keywords:** Peatlands, Forested wetlands, Carbon dioxide, Methane, Greenhouse gas, Soil flux

## Abstract

The Great Dismal Swamp, a freshwater forested peatland, has accumulated massive amounts of soil carbon since the postglacial period. Logging and draining have severely altered the hydrology and forest composition, leading to drier soils, accelerated oxidation, and vulnerability to disturbance. The once dominant Atlantic white cedar, cypress, and pocosin forest types are now fragmented, resulting in maple-gum forest communities replacing over half the remaining area. In order to determine the effect of environmental variabes on carbon emissions, this study observes 2 years of CO_2_ and CH_4_ soil flux, which will also help inform future management decisions. Soil emissions were measured using opaque, non-permanent chambers set into the soil. As soil moisture increased by 1 unit of soil moisture content, CH_4_ flux increased by 457 μg CH_4_–C/m^2^/h. As soil temperature increased by 1 °C, CO_2_ emissions increased by 5109 μg CO_2_–C/m^2^/h. The area of Atlantic white cedar in the study boundary has an average yearly flux of 8.6 metric tons (t) of carbon from CH_4_ and 3270 t of carbon from CO_2_; maple-gum has an average yearly flux of 923 t of carbon from CH_4_ and 59,843 t of carbon from CO_2_; pocosin has an average yearly flux of 431 t of carbon from CH_4_ and 15,899 t of carbon from CO_2_. Total Cha^−1^year^−1^ ranged from 1845 kg of Cha^−1^year^−1^ in maple-gum to 2024 kg Cha^−1^year^−1^ for Atlantic white cedar. These results show that soil carbon gas flux depends on soil moisture, temperature and forest type, which are affected by anthropogenic activities.

## Introduction

Forested peat wetlands store large quantities of carbon in the form of organic biomass (Anderson et al. 2016), largely in the root and soil pool (Powell and Day 1991). Human alteration of wetland hydrology can lead to drying of the soil, which leads to oxidation, changes in plant species composition, altered ecosystem health, increased fire risk, and potentially large emissions of greenhouse gases (GHG) (Reddy et al. 2015). Forest degradation and land use change are important contributors to climate change globally. This study aims to quantify the differential GHG fluxes of carbon from the soil matrices occurring in maple-gum (*Acer rubrum* and *Nyssa sylvatica*), Atlantic white cedar (*Chamaecyparis thyoides)*, and pocosin (*Pinus serotina*) habitats at the Great Dismal Swamp. The main drivers of carbon flux are generally soil and vegetation characteristics, including soil moisture and flooding, wetland or forest type, and soil chemistry; hence, we tested the hypotheses that the GHG flux of carbon differs between soils under maple-gum, Atlantic white cedar, and pocosin habitats, and that carbon flux is dependent on soil temperature and soil moisture. We also explore other possible variables contributing to differences in flux rates in the Great Dismal Swamp.

Some soil respiration has been measured in the Great Dismal Swamp, for example CH_4_ flux in 1980–1981, where waterlogged soil was found to be a CH_4_ source and soils during drought acted as a sink, while normally-dry forest soil did not act as a sink; temperature, season, and soil water content were used to determine CH_4_ flux within a maple-gum site in the Great Dismal Swamp (Harriss et al. 1982). Another study in Pocosin Lakes National Wildlife Refuge, a pocosin habitat relatively near the Great Dismal Swamp, consisting of natural, restored and degraded pocosins, found that phenolics present in the peats, which are found in higher concentrations in shrubs than herbaceous vegetation, protect against peat oxidation during short term droughts, mitigating the increase in CO_2_ emissions found in sphagnum wetland areas. Phenolics were found to be inversely related to soil respiration, protecting peat in shrub communities during droughts (Wang et al. 2015).

Many studies have quantified carbon flux in forest, peat and wetland soils (Bubier et al. 2003). Different variables affect carbon flux in different ecosystems. In subalpine forests of the Rocky Mountains in the US, leaf area index, soil nitrogen and tree height were found to account for much of the variability in positive total below ground carbon flux (Berryman et al. 2016). In a drained forested peatland in Finland, GHG flux (carbon uptake) was found to depend on season (irradiance and temperature), vapor pressure deficit and water table, but did not correlate with plant community composition or soil micro topography (Lohila et al. 2011). In the wet-dry topics of Australia, GHG soil flux was found to be controlled by soil moisture (Beringer et al. 2013). On a floodplain in the mid-Atlantic region of the US, carbon flux was found to depend on water-filled pore space and mass of deposited mineral sediment, clay fraction and particle size, temperature, pH, and soil redox (Batson et al. 2014). In open water and emergent vegetation, ebullition and diffusion of CH_4_ and CO_2_ flux were found to depend on season and wetland structure (McNicol et al. 2017). Different forests and wetlands can also act as a source or a sink. Snow-covered northern wetlands in China were found to act as a source or sink at different times of the year, depending on snow pack density, temperature, and type of wetland (Miao et al. 2012).

In this study, we measured carbon-based GHG emissions (CO_2_ and CH_4_) from soils present within three forested wetland habitat types that differ in peat chemistry, carbon density, and peat accretion (Drexler et al. 2017), and hydrologic patterns. We found CO_2_ and CH_4_ flux respond to changes in soil temperature and soil moisture as well as forest community type. The Great Dismal Swamp has experienced massive peat loss facilitated not only by chronic oxidation from perennially lower water tables, but also from dry-condition-induced fires that burn through thousands of years of peat deposition in relative short periods of time to reduce elevations, and further promote forest habitat shifts. The objectives of this study are to evaluate the consequence of these shifts on the fluxes of carbon-based GHGs and inform future management decisions.

## Methods

### Study design and site description

**Fig. 1:**
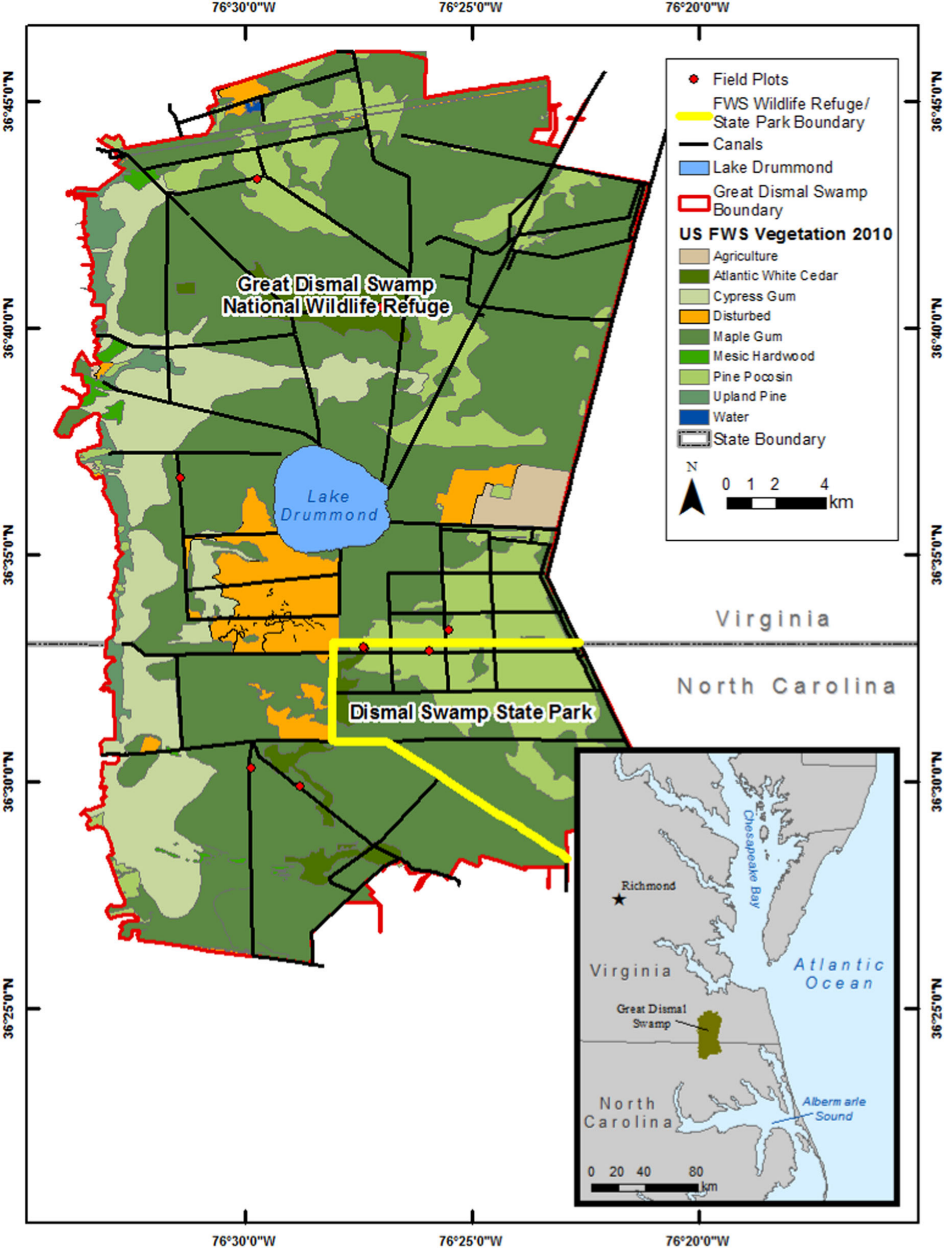
Map showing the Great Dismal Swamp with forest types as determined by The Natural Communities of Virginia, the locations of the nine study sites, and the location within the mid-Atlantic of the US (Fleming et al. 2001)

The Great Dismal Swamp is a freshwater forested wetland of over 54,000 ha located in southeastern Virginia and northeastern North Carolina, less than 64 km from the Atlantic coast. Currently, the US Fish and Wildlife Service manages the Great Dismal Swamp National Wildlife Refuge, while the small section in the southeast is managed by North Carolina Dismal Swamp State Park (Fig. 1). Before European settlement, the forested wetland was estimated to occupy over 404,000 ha in extent but has been reduced to its current size through anthropogenic pressures for development and clearing for agriculture (Laderman et al. 1989; Oaks and Whitehead 1979). There are still ~250 km of ditches and roads running through the Great Dismal Swamp (Barrd 2006), making hydrologic restoration a challenge. The native forest types once dominating the Great Dismal Swamp were baldcypress (*Taxodium distichum*) and Atlantic white cedar (*C. thyoides*) (Barrd 2006), with baldcypress in the wetter areas and Atlantic white cedar in the slightly higher areas (Brown and Atkinson 1999). Today, Atlantic white cedar and baldcypress still remain (Laing et al. 2011) in remnant populations along with pond pine (*P. serotina*), but red maple (*A. rubrum*), black gum (*N. sylvatica*) and sweet bay (*Liquidambar styraciflua*) (referred to as “maple-gum”) have become a major part of the contemporary forest composition, comprising over 60% of the current Great Dismal Swamp extent (Laderman et al. 1989) due to their resilience and ability to compete with other species in the new, drained conditions. Pine pocosin (hereafter, pocosin) is a type of fire-adapted wetland (Parthum et al. 2017) of the Atlantic coastal plain characterized by nutrient poor, often saturated (Kim et al. 2017) peat soils inhabited by pond pine (*P. serotina*) and loblolly pine (*Pinus taeda*), and a mix of dense shrubs (e.g., sweet pepperbush [*Clethra alnifolia*], inkberry [*Ilex glabra*] and greenbriar [*Smilax rotundifolia*]). Here, we focus on Atlantic white cedar and pocosin which are vulnerable to alternative succession by maple-gum.

Data were collected at three sites in each of the three forest types, using four sampling plots within each site, for a total of 9 sites and 36 sampling plots. Sites were chosen as good representatives of the target forest types in the Great Dismal Swamp, within accessibility constraints, based on mix of species within each forest type (maple-gum, Atlantic white cedar, and pocosin) (Jenkins et al. 2001), canopy cover, inundation and moisture regime, and disturbance. To determine the rate of soil CO_2_ and CH_4_ flux in the Great Dismal Swamp and the driving factors for these rates, two years of monthly CO_2_ and CH_4_ measurements were taken, as well as soil temperature at 10 cm and ambient air temperature. Gas fluxes (CO_2_ and CH_4_) were measured from chambers (adapted from Krauss and Whitbeck (2012) for use with tubes instead of syringes) set onto permanent bases, which were installed in each sampling plot, for a total of 12 chamber bases per forest type. Each site was visited once per month, with all sites visited within a 3–4 day period, and each base was sampled for 10 min each time. We also took monthly site-level moisture measurements of litter, soil of 0–5 cm depth, and soil of 5–10 cm depths within each site. Soil temperature at 10 cm was recorded from all sites over 2+ years using continuous loggers (model HOBO Pro v.2, Onset Computer Corp., Bourne, MA, USA) as well as manually during each sampling.

We minimized impact on the study site as much as possible by not walking near the chambers except to place the equipment, and by placing equipment from a bench to distribute the weight of the researcher. Any ebullition events under the chambers during the sample time were captured but none were noted. We also used an in situ portable cavity ring-down spectroscopy analyzer (Los Gatos Research Ultra Portable Greenhouse Gas Analyzer [UGGA], San Jose, California) to detect smaller concentrations of CH_4_ than the traditional gas chromatography technique (Christiansen et al. 2015), since CH_4_ is found in much lower concentrations than CO_2_ at these sites. Using the spectroscopy analyzer also allows for a short sampling time, which reduces the buildup of pressure inside the chamber which can reduce the diffusive flux of the system (Parkin et al. 2012) but still provides hundreds of data points at the sampling rate of over one measurement per second, enough to establish the flux rate.

### Gas flux measurements and ancillary data

#### Ancillary data

Each gas flux measuring chamber base was set and left for the duration of the study. Chamber bases measure 29.4 cm by 29.4 cm (864 cm^2^ area), and are 12.7 cm deep composed of straight sides forming an open top with a square trough in which to set the chamber top, which added 30.5 cm to the chamber height during sampling. The volume of the chamber when set into the base is 27.22 liters and was adjusted for reductions in volume due to surface water when flooded as necessary. This trough was filled with water before sampling so that the opaque chamber top and base form an air tight seal. Gas exchange was also blocked from below the chamber base by insertion to 12 cm into the soil, which was deep enough to avoid leakage during the sampling time (Rochette et al. 2008). While the chamber bases were permanent, the chamber tops were placed in the bases by hand during sampling only.

The UGGA intakes gas from a tube connected to the chamber top and returns it to the chamber after it is run through a cavity enhanced laser spectrometer. The UGGA sampling rate is once every 0.975 s and measurements are recorded in parts per million for both CO_2_ and CH_4_. The chamber volume was calculated by measuring the inner sides and top of the chamber, accounting for compaction and water volume. With no standing water, the chamber volume is ~27 liters. Water depth at each chamber was measured if applicable and used to adjust chamber volume. Tree age and diameter at breast height were measured once using tree cores and circumference.

The volume of gas in each chamber was sampled during the daytime each month by placing the chamber top into the bottom trough with the UGGA running. The chambers were left in place for 10 min and then removed and the analyzer was allowed to return to baseline by remaining open to the air for 4 min in between each sample. Air temperature sometimes varied by several degrees over the sampling period at each site. All measurements were during the day, between sun rise and sun set.

In order to minimize disturbance of the soil caused by the sampling process (Winton and Richardson 2016), wide footed stools were placed near each point before sampling and the chamber top was lowered onto each base from a plank set between the stools. This mitigates against negative measurement impacts (c.f., Winton et al. 2017). Leaving the chambers for one month—or greater in our case—before sampling also avoids errors based on soil disturbance from insertion (Muñoz et al. 2011). The analyzer was calibrated and maintained according to the manufacturer’s instructions.

### Statistical analysis

Statistical analysis includes basic statistics such as average emissions over time and by site, as well as linear regression which shows the relationship between the measured variables and gas flux, and a paired two sample *t*-test to show whether the forest classes statistically represent the same population based on measured variables. Ecosystem respiration is determined by calculating the slope of the increasing concentration of gas inside the chamber in ppm/0.975 s and converting that into CO_2_ or CH_4_ (and then into carbon) per square meter of ground surface. Unless otherwise noted, yearly data from this study is made up of measurements throughout all months of the year sampled, averaged and added together so that seasonal fluctuations are represented. Regression shows the relationships between the measured variables and the gas flux.

The paired two sample *t*-test of CO_2_ and CH_4_ fluxes in each forest type is used to show if two means come from the same statistical population. Linear regression is used to show the relationships between measured variables and gas flux.

## Results

Results include descriptive statistics showing the site characteristics and differences, linear regression showing variable effects on emissions, and a paired *t*-test to determine whether sample means indicate the same or different populations. CO_2_ net emissions, both averaged across all measurements and individually at the different sites, varied in response to the changing seasons and temperatures, as well as by forest type. Average CO_2_ net emissions in the peak growing season (April–September over 120.95 mg CO_2_–C/m^2^/h) was almost three times higher than CO_2_ flux in October–December (42.84 mg CO_2_–C/m^2^/h) and January–March (34.76 mg CO_2_–C/m^2^/h). CH_4_ flux also varied throughout the year but in response to soil moisture and other variables as well as season and temperature (Tables 1 and 2). See Gutenberg and Sleeter 2018.

**Table 1:** Basic statistics and distribution of measurements by site

	Carbon dioxide net emissions (μg CO_2_–C/m^2^/h)				Methane net emissions (μg CH_4_–C/m^2^/h)			
Site	Min	Max	STD	Mean	Min	Max	STD	Mean
C1	8.3 × 10^3^	3.0 × 10^5^	7.1 × 10^4^	9.4 × 10^4^	−1.7 × 10^1^	1.3 × 10^4^	1.4 × 10^3^	1.7 × 10^2^
C2	8.4 × 10^3^	2.8 × 10^5^	7.0 × 10^4^	8.7 × 10^4^	–3.5 × 10^1^	1.7 × 10^2^	2.3 × 10^1^	−2.7 × 10^0^
C3	1.1 × 10^4^	2.8 × 10^5^	5.5 × 10^4^	8.1 × 10^4^	−1.2 × 10^1^	1.0 × 10^3^	2.2 × 10^2^	1.0 × 10^2^
M1	1.0 × 10^4^	5.7 × 10^5^	1.0 × 10^5^	7.7 × 10^4^	−2.3 × 10^1^	1.2 × 10^2^	2.6 × 10^1^	3.4 × 10^0^
M2	7.0 × 10^3^	2.9 × 10^5^	6.8 × 10^4^	8.7 × 10^4^	−5.6 × 10^1^	5.7 × 10^2^	7.3 × 10^1^	2.3 × 10^1^
M3	7.3 × 10^3^	1.9 × 10^5^	3.3 × 10^4^	6.5 × 10^4^	−1.8 × 10^1^	2.0 × 10^4^	3.2 × 10^3^	1.3 × 10^3^
P1	7.8 × 10^3^	3.5 × 10^5^	6.5 × 10^4^	6.2 × 10^4^	−3.4 × 10^2^	5.7 × 10^4^	7.3 × 10^3^	2.3 × 10^3^
P2	1.5 × 10^4^	3.1 × 10^5^	6.6 × 10^4^	1.1 × 10^5^	−4.7 × 10^1^	3.6 × 10^2^	5.2 × 10^1^	−3.9 × 10^0^
P3	1.2 × 10^4^	2.7 × 10^5^	4.8 × 10^4^	8.7 × 10^4^	−2.3 × 10^1^	5.6 × 10^1^	1.6 × 10^1^	7.2 × 10^−1^

**Table 2:** Results of regression analysis of relationships between dependent and independent variables

	*P* values	Air temperature	Soil temperature	Soil moisture, litter layer	Soil moisture, 0–5 cm	Soil moisture, 5–10 cm
CO_2_	*All sites*	***<0.001***	***<0.001***	*0.081*	*0.074*	*0.211*
	Maple-gum	0.056	**<0.001**	0.104	0.758	0.357
	Pocosin	0.258	**<0.001**	**0.024**	**0.018**	0.567
	Cedar	**<0.001**	**0.001**	0.065	0.211	0.322
	Growing season (Apr.–Sept.)	**0.011**	**<0.001**	0.143	0.466	0.192
	Non growing season (Oct.–Mar.)	0.099	**0.001**	0.758	0.570	0.545
CH_4_	*All sites*	*0.518*	*0.266*	*0.388*	***0.027***	*0.102*
	Maple-gum	0.647	0.289	**0.013**	0.564	**0.007**
	Pocosin	0.458	0.474	0.351	**0.019**	0.206
	Cedar	0.576	0.542	0.579	0.574	0.641
	Growing season (Apr.–Sept.)	0.060	0.848	0.678	**0.011**	0.755
	Non growing season (Oct.–Mar.)	0.836	0.062	**0.012**	**0.048**	**0.011**

Values in bold are statistically significant at a 95% confidence level

The CO_2_ net emissions measurement distribution is positively skewed, with the greatest number of values between 7000 and 200,000 μg CO_2_–C/m^2^/h, with values ranging up to 567,897 μg CO_2_–C/m^2^/h. The distribution of CH_4_ flux measurements is also positively skewed, but to a greater extent with many very high outliers, with the greatest number of flux values between 0 and 50 μg CH_4_–C/m^2^/h, and a right tail of values ranging up to 56,686 μg CH_4_–C/m^2^/h. CO_2_ net emissions showed a more consistent range of values, whereas range of CH_4_ flux measurements varied by site (Table 1).

Linear regression shows that much of the variation in CO_2_ emissions is explained by changes in air and soil temperature and soil moisture from 0 to 5 cm. See Table 2 for actual results from all sites. Much of the variation in CH_4_ flux is explained by variation in water depth, soil moisture from 0 to 5 cm, and soil moisture from 5 to 10 cm, but less of the overall variation in CH_4_ flux is accounted for.

CO_2_ emissions across all the sites show a statistically significant relationship with air temperature (*P* < 0.001) and soil temperature (*P* < 0.001), as well as a relationship with soil moisture content (SMC) in the top 5 cm of soil (*P* = 0.074) and in the litter layer (*P* = 0.081) that is significant at a 90% confidence level. The slopes and *R*^2^ values for CO_2_ net emissions plotted against soil temperature varied by forest type. Atlantic white cedar sites had the best fit (*R*^2^ = 0.6419) followed by pocosin (*R*^2^ = 0.5588) and then maple-gum (*R*^2^ = 0.488). Increasing soil temperature caused a generally greater increase in CO_2_ net emissions on Atlantic white cedar sites than in pocosin or maple-gum sites, and a generally greater increase in CO_2_ net emissions in pocosin sites than in maple-gum sites (Atlantic white cedar slope 10,320 μg C/m^2^/h/°C, pocosin slope 8183 μg C/m^2^/h/°C, maple-gum slope 6693 μg C/m^2^/h/°C).

CH_4_ flux shows a statistically significant relationship with SMC in the top 5 cm of soil (*P* = 0.027), and a relationship with water depth (*P* = 0.090) which is statistically significant at a 90% confidence level. Average age of trees and average diameter of trees at breast height sampled at the sites do not show a significant relationship to CH_4_ (*P*-values 0.209 and 0.279, respectively) or CO_2_ (*P*-values 0.225 and 0.901, respectively) flux. Measuring 0–10 cm SMC was less helpful in a predictive sense than measuring 0–5 in both cases, suggesting a tight connection between gaseous carbon fluxes from the soil and condition in very upper soil horizon only. The relationship between CH_4_ flux and 0–5 cm or 5–10 cm soil moisture is not linear. The differences between the forest types are less pronounced when possible statistical outliers are removed, with outliers determined as values above the 3rd quartile value plus 1.5× the middle quartile range, and less than the 1st quartile value minus 1.5× the middle quartile range, in this case −83 to 120 μg CH_4_–C/m^2^/h.

Soil moisture varied by time and by site during this study, including fully saturated or flooded soil at most of the sites at some point during the year. Precipitation accounted for some of the water depth and soil moisture variation, but not all; the highest water depth measurements occurred after a high precipitation event, but some of the other higher water depth measurements appear to be unrelated to precipitation and may be due to water management. SMC varies spatially more than temporally, although soil moisture and water depth were generally higher in winter. SMC is defined as (wet weight–dry weight)/dry weight.

**Fig. 2:**
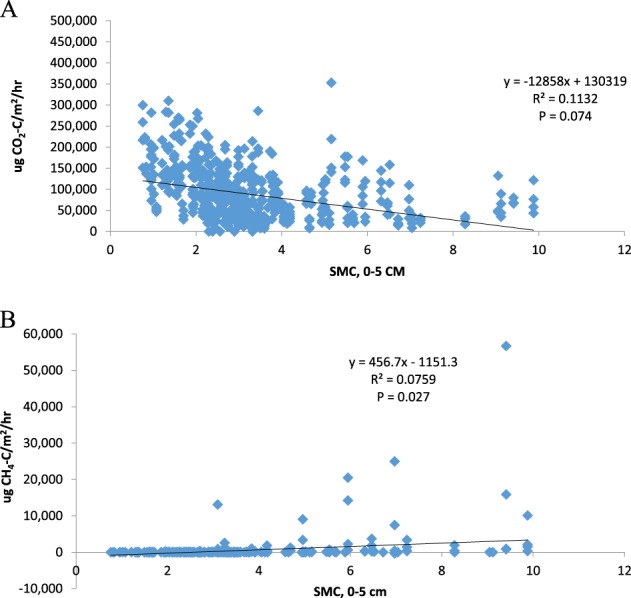
**a** Relationship between CO_2_ net emissions and soil moisture (0–5 cm), showing all plots sampled. **b** Relationship between CH_4_ flux and soil moisture (0–5 cm), showing all plots sampled

**Fig. 3:**
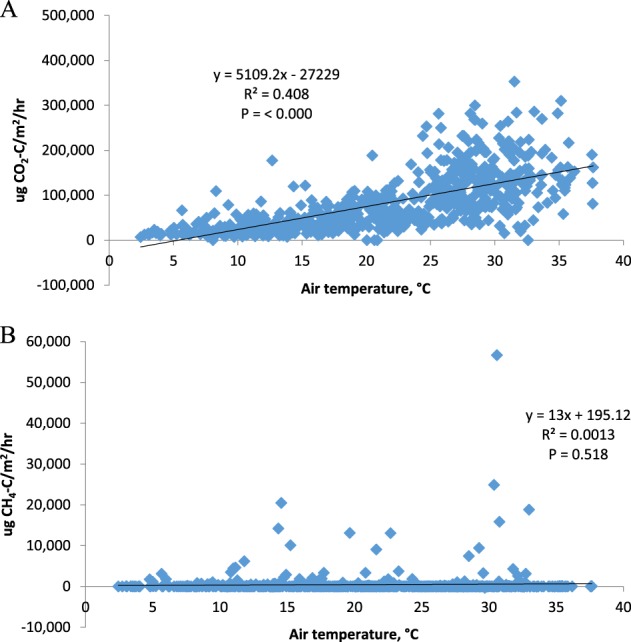
**a** Relationship between CO_2_ net emissions and air temperature. **b** Relationship between CH_4_ flux and air temperature

CO_2_ net emissions decreased as soil moisture increased (Fig. 2). CH_4_ flux increased with increasing soil moisture. Temperature varied by season during the duration of this study. CO_2_ net emissions increased with increasing temperature, and CH_4_ flux showed little relationship with temperature (Fig. 3). Over the year, CO_2_ net emissions vary with temperature but CH_4_ responded to soil moisture and water depth as well as temperature.

**Fig. 4:**
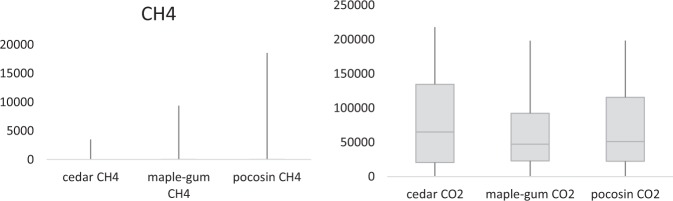
Boxplots

Statistical analysis showed that the three different forest communities studied have different rates of carbon gas flux and are therefore statistically separate populations in this regard (Table 3 and Fig. 4). The differences between Atlantic white cedar and the other two forest types in terms of CH_4_ flux were the only statistically significant values at a 95% confidence level, but all tests tended toward separation between the three populations in both measurements. Atlantic white cedar is the forest type with flux measurements most different from the other two, and maple-gum and pocosin are the populations with the most similar measurements.

**Table 3:** Paired *t*-test for two sample means showing that the three populations are likely separate, with statistically significant results in bold

CO_2_	Chance same pop.	T-stat	DF	*P* value	CH_4_	Chance same pop.	T-stat	DF	*P* value
Cedar and Maple-gum	16%	1.26	105	0.21	**Cedar and Maple-gum**	**3%**	**−2.01**	**80**	**0.05**
Maple-gum and Pocosin	38%	−0.90	112	0.37	Maple-gum and Pocosin	16%	−1.02	74	0.31
Cedar and Pocosin	27%	0.40	106	0.69	**Cedar and Pocosin**	**2%**	**−2.02**	**57**	**0.05**

## Discussion

### Drivers of flux

The results show that forest type has an effect on flux, in addition to the effects of moisture and temperature. Drivers behind soil respiration and carbon gas flux in other studies include ground water or surface water level and source, vegetation type, water-filled pore space, restoration status, temperature and pH. In this study, soil moisture and temperature were the main drivers for CH_4_ and CO_2_ flux, respectively, with some influence from forest type (Figs 2, 3 and Table 3).

In this study, soil moisture from 0–5 cm deep was more highly correlated to CH_4_ flux than soil moisture 5–10 cm deep, indicating the relationship to depth to ground water in other studies may be a measure of field capacity or water retention of the soil and time since last rewetting. CO_2_ net emissions decreasing as soil moisture increases is expected and may be related to the biogeochemical chain of electron acceptors as oxygen is depleted in flooded conditions. This may also be related to reduced metabolic activity and increased soil moisture in the colder months. Refuge management activity affects water level in the ditches and therefore depth of the water table, especially closer to the roads and ditches. This was taken into account by the water depth variable. Of the sites sampled in this study, two were flooded frequently, the northernmost pocosin site and the northernmost maple-gum site. The Atlantic white cedar and pocosin sites that are closest to the highest concentration of ditches were least often flooded.

#### Comparison with other studies—CO_2_

Net CO_2_ emissions in the Great Dismal Swamp in this study was 740 g C/m^2^/year for cedar, 684 g C/m^2^/year for maple-gum, and 711 g C/m^2^/year for pocosin, or 7397 kg C/ha/year for cedar, 6844 kg C/ha/year for maple-gum, and 7113 kg C/ha/year for pocosin. The individual sites ranged from the lowest CO_2_ fluxes in the wettest, most frequently inundated sites (low of 575 g C/m^2^/year at the wettest maple-gum site), to the highest in the dryer sites (817 g C/m^2^/year at a dry pocosin site). These CO_2_ measurements are within the ranges reported in the studies below.

Other studies of soil respiration in forests and wetlands as well as in laboratory incubation experiments have reported a wide range of values, mainly higher than the values found in this study. These measurements were either reported in, or were converted to, grams of carbon, per square meter, per year (g C/m^2^/year). For CO_2_ net emissions, high values were seen in a Canadian peatland, with very high values when the water table was 70 cm below the surface (239,805.00–341,540.45 g C–CO_2_/m^2^/year), and still fairly high when the water table was 10 cm above the surface (10,900–18,167 g C–CO_2_/m^2^/year) (Moore and Knowles 1989). Moderate levels of CO_2_ flux were seen in North Carolina peatlands and pocosins, with short pocosin ranging from 438 to 1314 g C/m^2^/year, tall pocosin (which is more similar to the pocosin in the Great Dismal Swamp) ranging from 788 to 1095 g C/m^2^/year, and maple-gum ranging from 657 to 2190 g C/m^2^/year (Bridgham and Richardson 1992). Peak soil respiration in the subalpine Rocky Mountains was also moderately high at 1654 g C/m^2^/year (Berryman et al. 2016). A floodplain in Virginia saw similar levels of soil respiration, with 1091 g C–CO_2_/m^2^/year (Batson et al. 2014). Chambers in the water of a restored wetland in California also showed similar levels of 915 g C–CO_2_/m^2^/year (McNicol et al. 2017). Low levels of CO_2_ soil flux were seen in peatlands during snow cover in the winter in China, with 3–12 g C–CO_2_/m^2^/year in the peat and 20 g C–CO_2_/m^2^/year in a marsh (Miao et al. 2012). A natural site in Pocosin Lakes National Wildlife Refuge measured only 14 g C–CO_2_/m^2^/year (Wang et al. 2015). Peatland in Australia and forestry drained peatland in Finland showed net uptake of CO_2_, −308 g C–CO_2_/m^2^/year (Beringer et al. 2013) and −237 g C–CO_2_/m^2^/year (Lohila et al. 2011) respectively.

#### Comparison with other studies - CH_4_

CH_4_ flux in the Great Dismal Swamp in this study was 0.05 g C/m^2^/year for the cedar forest type, 1.29 g C/m^2^/year for maple-gum, and 3.81 g C/m^2^/year for pocosin. This is lower than the 1982 CH_4_ measurements shown below, which were taken over a 17-month period in maple-gum forest cover, but more in line with the low to moderate results in other study areas.

For CH_4_, Great Dismal Swamp measurements in the maple-gum forest type from 1982 showed high flux rates of 130 g C–CH_4_/m^2^/year to 1968 g C–CH4/m^2^/year (Harriss et al. 1982). The high CH_4_ values may be due to ebullition from pockets in the soil air space or below the water table or water surface in the case of flooded conditions. Other measurements are relatively low; 2.9 g C–CH_4_/m^2^/year in restored California wetland water chambers (McNicol et al. 2017), 0.01–0.04 g C–CH_4_/m^2^/year in snow-covered peatlands in China (Miao et al. 2012), 7.67 g C–CH_4_/m^2^/year in an inundated fen in Canada, and 0.19 g C–CH_4_/m^2^/year in an inundated bog in Canada (Moore and Knowles 1989).

#### Total area

The forest cover of the Great Dismal Swamp is 61% maple-gum, 15% pocosin, 12% cypress-gum, 3% Atlantic white cedar and 9% other with a total study area of 54,000 ha (Fleming et al. 2001). Using these figures as a guideline, the total area of Atlantic white cedar in the study area (1620 ha) has an average yearly flux of 0.75 metric tons carbon from CH_4_ and 11,983 metric tons of carbon from CO_2_. The total area of maple-gum in the study area (32,940 ha) has an average yearly flux of 425 metric tons of carbon from CH_4_ and 225,457 metric tons of carbon from CO_2_. The total area of pocosin in the study area (8100 ha) has an average yearly flux of 309 metric tons of carbon from CH_4_ and 57,617 metric tons of carbon from CO_2_. The total yearly carbon loss (not including uptake due to plant productivity and carbon burial) from the study area made up of these three forest types (54,000 ha minus the 21% that is cypress-gum or other is 42,660 ha) would then be 295,792 metric tons of carbon per year, from soil flux alone.

### Increased sampling efficiencies

In an attempt to minimize error due to sampling, the techniques were designed to minimize researcher impact on the system: keeping the bases in place over the duration of the study; not stepping on the ground near the chambers when avoidable; leaving the sites as natural as possible; and keeping field equipment away from the chambers by using 12 foot long fluorinated ethylene propylene tubes. Despite these precautions, it is possible that motion above the ground could have caused some ebullition of CH_4_ from below ground during sampling. For example, bubbles rising through the standing water were often visible when approaching flooded sites. However, these small bubbles seemed to be restricted to the paths used for walking and were not seen in the chamber areas.

Hutchinson and Livingston (2001) provide several recommendations for reducing error during chamber sampling—an air tight chamber base, in our case achieved using the water trough seal; sufficient chamber base installation depth, based on soil porosity and sampling time. Our depth of 12.7 cm deep is easily sufficient according to that study’s calculations, given a short sampling time of 10 min, even at the highest calculated porosity (requiring at least 8.6 cm depth). Their recommendation that all chambers include a vent near ground level aims to reduce error due to sudden changes in pressure during sampling—for example, when the chamber top is placed and when gas is extracted as a sample. However, since we used continuous sampling, there were no disturbances to pressure leading to the disturbances that they observed in their study. Also, the short sampling time and reflective white chamber tops eliminate risk of pressure change due to increasing temperature inside the chamber as compared with the outside temperature. Additionally, our continuous sampling allows us to see if any large disturbances occur in real time. Some small deviation from the overall linear trends at the very beginning of sampling may have been due to the pressure change of placing the chamber top.

Smith and Dobbie (2001), although studying N_2_O soil emissions in agricultural land, found mostly statistically insignificant differences between sampling several days apart interpolating, and sampling every 8 h, as well as between samplings at different times of the day. Rochette and Eriksen-Hamel (2008) found that many methods were sufficient for N_2_O treatment comparison, but insufficient for comparison with other studies at other sites due to the limitations of their physical techniques. However, our study avoids many of these common pitfalls; although our chambers are unvented and uninsulated, our deployment duration was only 10 min. In addition, we had sufficient insertion depth, chamber height greater than 10 cm, no sample handling or storage, no use of plastic syringes, and no delay between sampling and analysis. We did not, however, use quality control gas standards as suggested in Rochette and Eriksen-Hamel (2008) but did follow the manufacturer’s instructions for maintenance at a greater than 99% accuracy level (Los Gatos Research, Inc. specifications 2014).

### Implications of the study

Since soil moisture is responsive in part to manageable conditions, there is the possibility of management activities influencing future carbon gas flux. Prior to the 1970’s, when the Great Dismal Swamp National Wildlife Refuge was established, land use decisions (i.e. ditch construction and forestry) led to drier soil conditions and ecosystem vulnerability, which, given these findings, could have not only reduced CH_4_ flux, but also changed the characteristics of the soil and plant communities in addition to the changes caused directly by harvesting select species of timber. For example, fire susceptibility in terms of ignition success, burn depth and total combustion depends on factors including soil moisture, bulk density, organic matter component and species composition (Benscoter et al. 2011). In the Great Dismal Swamp, wetter areas with higher mean water levels were found to have thicker peat and higher species richness than drier areas, and while conditions in wetter areas did not meet fire risk conditions, drier areas were found to be always at risk of burning (Schulte 2017). CH_4_ flux, however, is a small fraction of net carbon flux. Future rewetting of the swamp may change plant and microbial communities once again, favoring wetland species that are tolerant of frequent flooding, overall moister conditions, and anoxic root zones (Ausec et al. 2009; Hartman et al. 2008). This may lead to increased CH_4_ flux and decreased CO_2_ net emissions. Rewetting may also cool the soil through evapotranspiration, further reducing CO_2_ net emissions and possibly mitigating some temperature increase due to changing global temperatures.

The limitations of this project suggest opportunities for future study such as sampling over greater temporal resolution or sampling over the length of a 24-h period to determine the effect of sunlight hours to see whether the same patterns persist. Such measurement disparity can be seen when eddy covariance measurements occurring over 24-h periods are compared with chamber methods; CH_4_ fluxes were 2–4 times higher from chambers (Krauss et al. 2016). Sampling differences from one day to the next with minimal changes in temperature or moisture would also be informative, since sampling each chamber only once a month means we do not have a full seasonal picture either. Sampling before, during and after a weather event would also be useful and could show the effect of rising ground water on flux as air is replaced with water in soil air pockets. Since surface soil moisture and temperature can be measured remotely using satellite data, it may be possible to model GHG flux throughout the refuge.

## Conclusion

CH_4_ flux increased as temperature increased for pocosin, but decreased with temperature for cedar and maple. All of the CH_4_ fluxes increased as soil moisture increased. On average, as soil moisture increased by one unit of SMC, CH_4_ flux increased by 457 μg C–CH_4_/m^2^/h (Fig. 2). On average, as temperature increased by 1 °C, CO_2_ flux increased by 5109 μg C–CO_2_ m^2^/h (Fig. 3). Cedar average CH_4_ flux was significantly different from both maple and pocosin. These results show that soil carbon gas flux depends on soil moisture, temperature, and forest type, all as affected by anthropogenic activities in these peatlands.

Overall, CO_2_ net emissions occurred at much higher concentrations than CH_4_ flux in the Great Dismal Swamp. CH_4_ uptake sometimes outpaced production, but the soil was usually a net source, while CO_2_ net emissions always showed a net source. Different forest types showed somewhat different trends. CO_2_ was primarily associated with soil temperature, and CH_4_ flux was primarily associated with soil moisture in the top 5 cm (surface soil moisture). This information is relevant to assessing the implications of changing management decisions as habitat in the refuge is being restored. Although the US Fish and Wildlife Service does not manage for carbon sequestration, it is valuable to understand how changing hydrologic regimes that increase or decrease soil moisture may impact carbon balance in addition to habitat and other management goals (Sleeter et al. 2017).

This study shows the relationship between surface soil moisture and temperature, and gas flux. More variables could be studied to determine their relationship with soil carbon gas flux. The forests in the Great Dismal Swamp have been managed for centuries, which has very likely influenced current conditions including hydrology and soil conditions, since the peat soil is composed of organic matter accumulated over thousands of years. Topography, water flow, and soil nutrients would also play a role. While this study looked at sites situated in representative examples of three different forest types, more replication of these sites in different conditions could provide more information on gas flux in different hydrologic regimes, disturbed conditions, growth stages and tree maturities, and combinations of these factors.
